# Small Cages with Insect Couples Provide a Simple Method for a Preliminary Assessment of Mating Disruption

**DOI:** 10.1100/2012/960468

**Published:** 2012-05-03

**Authors:** Françoise Briand, Patrick M. Guerin, Pierre-Joseph Charmillot, Patrik Kehrli

**Affiliations:** ^1^Station de recherche Agroscope Changins, Wädenswil ACW, CP 1012, 1260 Nyon, Switzerland; ^2^Institute of Biology, University of Neuchâtel, Rue Emile-Argand 11, 2009 Neuchâtel, Switzerland

## Abstract

Mating disruption by sex pheromones is a sustainable, effective and widely used pest management scheme. A drawback of this technique is its challenging assessment of effectiveness in the field (e.g., spatial scale, pest density). The aim of this work was to facilitate the evaluation of field-deployed pheromone dispensers. We tested the suitability of small insect field cages for a pre-evaluation of the impact of sex pheromones on mating using the grape moths *Eupoecilia ambiguella* and *Lobesia botrana*, two major pests in vineyards. Cages consisted of a cubic metal frame of 35 cm sides, which was covered with a mosquito net of 1500 **μ**m mesh size. Cages were installed in the centre of pheromone-treated and untreated vineyards. In several trials, 1 to 20 couples of grape moths per cage were released for one to three nights. The proportion of mated females was between 15 to 70% lower in pheromone-treated compared to untreated vineyards. Overall, the exposure of eight couples for one night was adequate for comparing different control schemes. Small cages may therefore provide a fast and cheap method to compare the effectiveness of pheromone dispensers under standardised semi-field conditions and may help predict the value of setting-up large-scale field trials.

## 1. Introduction

The use of synthetic insecticides since the end of the Second World War has served to increase considerably the world's food production, but these pesticides also compromise the environment and human health [1–3]. Moreover, their extensive use favours the development of resistant insect pests and harms beneficial insects, occasionally resulting in the outbreak of secondary pest species [4]. Social awareness of the drawbacks of these classical insecticides in the early sixties urged the biotechnological industry to develop safer and more ecologically friendly alternatives [5, 6]. One of these alternatives was the interfering with pheromone-mediated mate-finding systems [7, 8]. Gaston et al. [9] were one of the first to confirm that premating communication between sexes could be disrupted by releasing synthetic sex pheromones into the atmosphere. The diffusion of a pest's pheromone impairs the ability of males to locate sexually receptive females and so reduce or even prevent mating [10, 11]. Today, the validity of manipulating and interfering with insect olfactory communication systems via the use of synthetic pheromones has been demonstrated for many insect species, and mating disruption has been established as an effective and sustainable integrated pest management measure in a broad range of cropping systems [7, 12]. For example, mating disruption has been implemented to control the codling moth *Cydia pomonella* in apple and pear orchards, the pink bollworm *Pectinophora gossypiella* in cotton and the grape moths *Eupoecilia ambiguella* and *Lobesia botrana* in vineyards [7]. Overall, mating disruption is the result of behaviour and physiological effects, which can be classified as completive attraction, camouflage, and desensitisation [8, 13, 14]. 

A downside of mating disruption is the laborious development process of pheromone dispensers as well as the challenging assessment of its effectiveness [15]. Electrophysiological responses of antennal receptor neurons are a useful first step to identify the basic chemical components of sex pheromones. After identification, the components need to be synthesised in the laboratory and their attractiveness has to be tested in baited traps deployed in the field. The following step is to evaluate the effectiveness of the developed pheromone dispensers for mating disruption in the actual crop. This is usually done by assessing pest densities or crop damage in commercial fields treated with pheromone dispensers compared to comparable fields that remained untreated. To achieve reliable results, pheromone-treated fields should be of a minimal size, quite frequently up to three hectares [16, 17]. Because pheromone dispensers are ideally evaluated on a sizeable scale, the environmental conditions prevailing in different fields used for tests are rarely alike. The abundance of pests, crop varieties, cultural practices, microclimate, and soil can vary significantly between treated and untreated plots. Obtaining statistically sound data requires therefore many independent repetitions, which is demanding in terms of time, space, and costs.

Several alternative methods have been proposed for a preliminary assessment of pheromone dispensers for mating disruption. One of these is the exposure of tethered virgin females in pheromone-treated and untreated fields. After a defined period, exposed females are collected and dissected in order to determine the presence of spermatophores or sperms [18]. However, females are exposed in a quite artificial manner, where natural courtship behaviour is frequently compromised. In addition, these defenceless tethered females are regularly consumed by predators [19]. With the aim of testing the effectiveness of pheromone dispensers in a more natural setup, Doye and Koch [15] proposed the use of large insect enclosure field cages (e.g., 2.3 × 2.3 × 1.6 m). These cages were set up in pheromone-treated and untreated fields, and a defined number of males was released within each cage. To assess the effectiveness of pheromone dispensers, females were exposed in small netted boxes in standard delta-traps and the number of males recaptured in the two treatments compared. A similar approach was also taken by several other authors [20–25]. These authors exposed a defined number of insect couples in field cages, but their cages were significantly smaller (e.g., between 0.001 and 0.2 m^3^) and the effectiveness of mating disruption was evaluated by dissecting exposed females to assess their mating status.

Even though such small insect field cages were used in the past to assess mating disruption [20–25] or at least the noncompletive mechanisms mediating disruption [13], they are not commonly employed for testing newly developed pheromone dispensers. A refinement of these small cages may therefore provide a welcome asset to the biotechnological industry in order to obtain preliminary and relatively rapid indications of a pheromone dispenser's effectiveness in the field under standardised conditions. With this in mind, we made use of the European vine moth, *Lobesia botrana* (Den. & Schiff.), and the grape berry moth, *Eupoecilia ambiguella* (Hübner). These two tortricid moths coexist over a large part of Europe where they represent the two major lepidopteran pest insects of grapevines (*Vitis vinifera L.*). Whereas adults of *L. botrana* are crepuscular, *E. ambiguella* is nocturnal [26]. Larvae primarily feed on shoots, flowers as well as fruits, and wounded berries are more vulnerable to the growth of pathogenic fungi such as botrytis (*Botrytis cinerea*) [27]. In general, these two moths are controlled by the application of insecticides, but mating disruption has been widely implemented over the last decade, and, today, there are several types of pheromone dispensers available on the market [8, 28, 29]. In this study, we present a generic approach on how to construct and test small field cages that permit an initial evaluation of newly developed pheromone dispensers under standardised semifield conditions. The first step in the development of such a field cage consisted of the construction of a prototype. In a second step, the efficiency of the prototype was examined and its design was refined. Finally, the effects of varying the number of exposed insects and the duration of their exposure were examined.

## 2. Materials and Methods

### 2.1. Study Site

Field trials were conducted in three different vineyards around Nyon, Switzerland. The distance between them was between 500 and 1100 meters, and they were all three about 3 hectares in size.

### 2.2. Pheromone Dispensers

Commercially available Isonet-LE and Isonet L-Plus pheromone dispensers manufactured by Shin-Etsu Chemical Co. Ltd. (Tokyo, Japan) were used. Isonet-LE dispensers contained a total of 182 mg (*E*, *Z*)-7,9-dodecadienyl acetate (=E7, Z9-12:Ac) and 182 mg (*Z*)-9-dodecenyl acetate (=Z9-12:Ac), the principal components of the pheromone blend of *L. botrana* and *E. ambiguella*, respectively. Isonet L-Plus dispensers contained 159 mg of E7, Z9-12:Ac but only 20.4 mg Z9-12:Ac. Both dispensers are registered in Switzerland against *E. ambiguella* and *L. botrana,* and they were both deployed at the recommended density of 500 per hectare.

### 2.3. Insects

*Lobesia botrana* and *E. ambiguella *used in this study originated from a permanent laboratory culture at Agroscope Changins-Wädenswil. Moths were reared on a semiartificial diet [30] in a climate chamber (16 : 8 h L : D cycle, 70 ± 10% RH and 22°C). Ten days after egg hatch, corrugated cardboard strips were placed in the rearing boxes (19 × 9 × 8 cm) to afford larvae a place to pupate. Pupae were sexed, and males and females were separated. After emergence, adults were transferred into cylindrical plastic boxes (*Ø* = 10.5 cm, *h* = 15 cm), where they had free access to a 10% sucrose solution. For the next two to four days, moths were stored in a room at ambient temperature and natural photoperiod. After this, couples of *L. botrana* or* E. ambiguella* aged between 3 and 5 days old were exposed in the field cages. Moths were always released into cages at the end of the afternoon and were recovered in the morning. Moths collected were killed and placed in 70% ethanol. Insect exposures were only made when night temperature was above 13°C and no precipitation was forecasted [26].

### 2.4. Field Cages Deployed with a Pheromone Dispenser

The goal of the first field trial was (1) to examine if grape moths mate inside of small insect field cages and (2) to test if these cages are suited to measure the effect of sex pheromones on mating. The core of these field cages consisted of a cubic metal frame of 35 cm side length. The frame was covered with a cotton tissue (800 *μ*m mesh). Cages were opened and closed by knotting of the tissue on one side of the cage, and they were set up in three differently treated vineyards. The first two vineyards were either equipped with Isonet-LE or Isonet L-Plus pheromone dispensers and the third vineyard served as an untreated reference. Two field cages were set up per vineyard in the middle of the foliage about one meter from the ground. In addition, a pheromone dispenser was fixed in the centre of each cage in the two pheromone-treated vineyards. Between July and August 2006, five couples of *L. botrana* or *E. ambiguella* were exposed simultaneously in these field cages for one night. All three treatments were repeated over 12 different nights for *L. botrana* and 10 different nights for *E. ambiguella*.

### 2.5. Field Cages Deployed without a Pheromone Dispenser

The aim of the second field trial conducted in the summer 2006 was to test if field cages not containing dispensers were an effective means of comparing mating disruption in the field. The trial was set up in the same vineyards and in the same cages (35 × 35 × 35 cm, mesh size = 800 *μ*m) as described above. However, no pheromone dispensers were placed in the cages installed in the vineyards treated with the Isonet-LE or Isonet L-Plus pheromone dispensers and the nearest dispenser was 4 meters away. Once again five couples of *L. botrana* or *E. ambiguella* were exposed in the cages for one night. Each treatment was repeated between 5 to 11 nights for *L. botrana* and 4 nights for *E. ambiguella*.

### 2.6. Refinement of Field Cage Tissue

With the aim to improve the flow of air into the field cages, the mesh size of the tissue covering the cages was increased. The cubic metal frames of 35 cm side length were covered with a polyester mosquito net of 1500 *μ*m mesh size. In addition, cages were modified for access on one side by a Velcro strip. The refined cages were set up in three vineyards. Two vineyards were either equipped with Isonet-LE or Isonet L-Plus pheromone dispensers and the third vineyard served as reference. Two field cages were put up in the middle of the foliage per vineyard, and no pheromone dispensers were deployed within the cages. Once again dispensers were at least 4 meters away of the cages. From June to August 2007, five couples of *L. botrana* or *E. ambiguella* were exposed in these refined field cages for a single night. Treatments were repeated on 28 and 26 different nights for *L. botrana* and *E. ambiguella*, respectively.

### 2.7. Refinement of the Duration of Insect Exposure

For a further refinement of the insect field cage method, the optimal length of time of insect exposure was tested. In 2008, cages of the same type as used in the previous year (35 × 35 × 35 cm, mesh size = 1500 *μ*m) were set up in three differently treated vineyards, that is, two vineyards either equipped with Isonet-LE or Isonet L-Plus dispensers and a third served as reference. Six field cages were installed in each vineyard, and no pheromone dispensers were deployed within cages. Only *E. ambiguella* was tested. Between May and June 2008, five couples were exposed in cages for either one, two, or three nights. Each treatment was repeated five times.

### 2.8. Refinement of the Number of Insects Exposed

To find the optimal insect density within field cages, 1, 2, 5, 8, 12, and 20 couples of *E. ambiguella* were exposed within a single cage. For a more accurate assessment of the actual mating success at the two lowest insect densities, one and two couples of *E. ambiguella* were exposed simultaneously in three and two cages, respectively. Thereafter, data were pooled and the arithmetic means of the proportion of mated females were calculated for each simultaneously exposed density. The experiment was conducted in the same cages (35 × 35 × 35 cm, mesh size = 1500 *μ*m, without any dispensers within cages) and in the same vineyards (Isonet-LE, Isonet L-Plus, and reference) as in the previous trials for assessing the optimal duration of insect exposure. Once again only *E. ambiguella* was tested and couples were exposed for one night. Between May and June 2008, the six treatments were repeated four times.

### 2.9. Assessment of Mating Disruption

To determine the mating status of preserved females, their bursa copulatris were dissected to confirm the presence or absence of spermatophores. To extract the bursa copulatris, the female abdomen was degreased in a 12% KOH solution of 80°C. This process took 5 and 10 minutes for *L. botrana* and *E. ambiguella*, respectively. Thereafter, the abdomen was immersed in demineralised water for 10 minutes and then rinsed for 5 minutes with 70% ethanol. The bursa copulatris was carefully extracted from the degreased abdomen under the binocular. When at least a single spermatophore was present, females were classified as mated.

### 2.10. Statistical Analysis

Data for *L. botrana* and *E. ambiguella* were analysed separately. The proportion of females mated per treatment and replicate was arcsine-square-root-transformed and treated as the dependent variable, whereas date of exposure, pest control scheme (=Isonet-LE, Isonet L-Plus, and reference), and duration of exposure were treated as nominal independent variables. Except for the experiment examining the number of released insect couples, all trials were analysed separately by either a one-, two-, or three-way ANOVA. The experiment assessing the effect of insect density in field cages was analysed by a two-way ANCOVA. The proportion of females mated was the dependent variable, whereas date of exposure and type of pheromone dispenser were treated as factors and number of exposed couples was included in the model as a covariate. For all statistical tests, means of pest control scheme and duration of exposure were compared pairwise with Tukey HSD post hoc tests. The fulfilment of model assumptions was checked by visual inspection of the distribution of residuals for every statistical test conducted. Throughout the text, means are given ± SD.

## 3. Results

### 3.1. Field Cages Deployed with a Pheromone Dispenser

**Figure 1:**
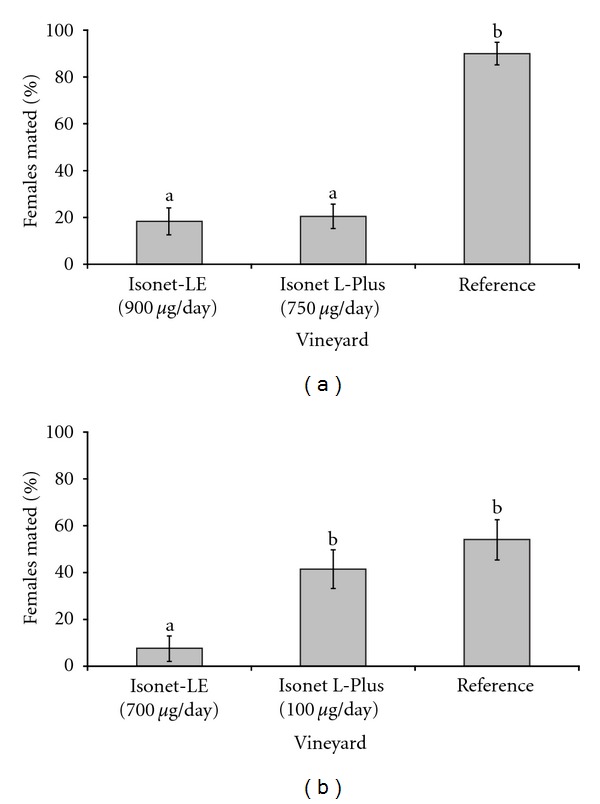
Percentage of mated (a) *L. botrana* and (b) *E. ambiguella *females in field cages (mesh size = 800 *μ*m) containing a pheromone dispenser. The notation *μ*g/day represents the approximate daily emission rate of pheromone dispensers for (a) *L. botrana* and (b) *E. ambiguella*. Pest control schemes with different letters are significantly different (*P* < 0.05); data are untransformed; bars = 1 SE.

One hundred and seventy-eight of the 180 exposed *L. botrana* females were recovered and dissected. The two-way ANOVA revealed that the date of exposure had no effect on mating (*F*_11;  22_ = 1.62, *P* = 0.160), but there was a significant effect of pest control schemes (*F*_2;  22_ = 71.82, *P* < 0.001, Figure 1(a)). Significantly more females were mated in the reference vineyard compared to the two vineyards equipped with pheromone dispensers. However, there was no statistical difference in the effectiveness of Isonet-LE and Isonet L-Plus dispensers. For *E. ambiguella*, 134 of the 135 exposed females were recovered. Whereas the date of exposure only tended to affect mating (*F*_9;  15_ = 2.15, *P* = 0.092), the type of control scheme had a significant effect (*F*_2;  15_ = 9.97, *P* = 0.002, Figure 1(b)). Significantly fewer females were mated in the Isonet-LE-equipped vineyard than in the reference or the Isonet L-Plus-treated vineyard, and there was no significant difference between the reference vineyard and the one treated with Isonet L-Plus dispensers emitting a lower amount of Z9-12:Ac. Overall, 99% of exposed females were recovered and 73.6 ± 27.9% of females exposed in the reference vineyard were mated. Thus, grape moths are capable to mate inside field cages. Moreover, cages seemed also to be suited for assessing the effectiveness of pheromone dispensers.

### 3.2. Field Cages Deployed without a Pheromone Dispenser

97% of *L. botrana* and *E. ambiguella *females exposed (*N* = 195) were recovered and dissected. The one-way ANOVA showed that pest control schemes had a significant effect on the mating status of *L. botrana* (*F*_2;  24_ = 12.82, *P* < 0.001). The proportion of females mated was significantly higher in the reference vineyard than in the vineyards equipped with either the Isonet-LE or Isonet L-Plus dispensers, that is, 89.1 ± 13.8%, 60.0 ± 14.1%, and 64.1 ± 13.2%, respectively. The two mating disruption treatments did not affect mating by *E. ambiguella* (*F*_2;  9_ = 0.43, *P* = 0.665). The proportion of mated females was 42.5 ± 43.5%, 22.5 ± 26.3%, and 26.3 ± 25.0% in the reference, Isonet-LE, and Isonet L-Plus treated vineyard, respectively. Without a pheromone dispenser in cages covered with a dense cotton tissue, mating in *E. ambiguella* was less disrupted.

### 3.3. Refinement of Field Cage Tissue

**Figure 2:**
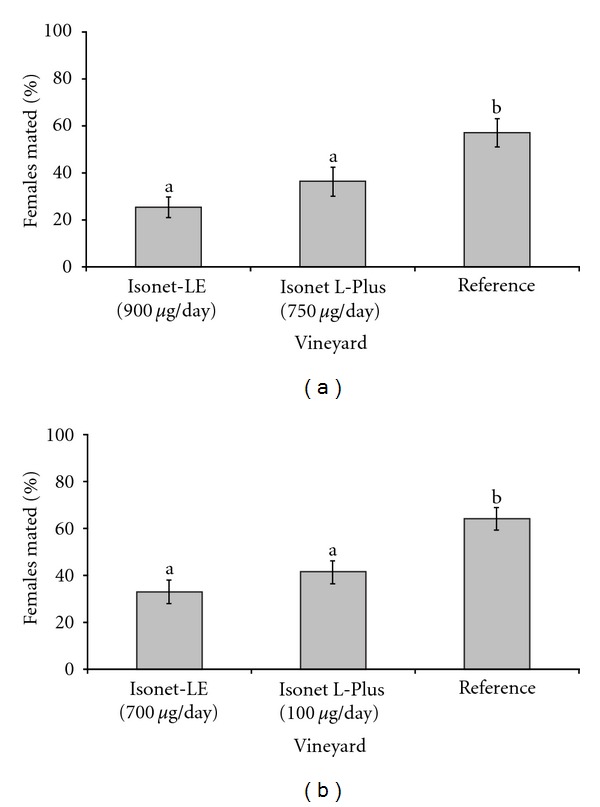
Percentage of mated (a) *L. botrana* and (b) *E. ambiguella *females in field cages (mesh size = 1500 *μ*m) containing no pheromone dispenser. The notation *μ*g/day represents approximately the daily emission rate of pheromone dispensers for (a) *L. botrana* and (b) *E. ambiguella*. Pest control schemes with different letters are significantly different (*P* < 0.01); data are untransformed; bars = 1 SE.

More than 95% of females exposed (*N* = 810) were recovered and dissected from the cages covered with the mosquito net of 1500 *μ*m mesh size. The two-way ANOVA showed that date of exposure (*F*_27;  54_ = 5.93, *P* < 0.001) as well as pest control schemes (*F*_2;  54_ = 23.63, *P* < 0.001, Figure 2(a)) had a significant effect on the mating status of *L. botrana*. The proportion of mated females was significantly higher in the reference vineyard compared to the vineyards equipped with either Isonet-LE or Isonet L-Plus dispensers. There was no statistical difference between the two dispenser types. For *E. ambiguella*, the date of exposure only tended to affect mating (*F*_25;  50_ = 1.53, *P* = 0.099), whereas the type of control scheme had a significant effect (*F*_2;  50_ = 13.48, *P* < 0.001, Figure 2(b)). Significantly fewer females were mated in the two pheromone-treated vineyards than in the reference. Furthermore, there was no statistical difference in the effectiveness between the lower charged Isonet L-Plus and the more heavily charged Isonet-LE dispensers.

### 3.4. Refinement of the Duration of Insect Exposure

**Figure 3:**
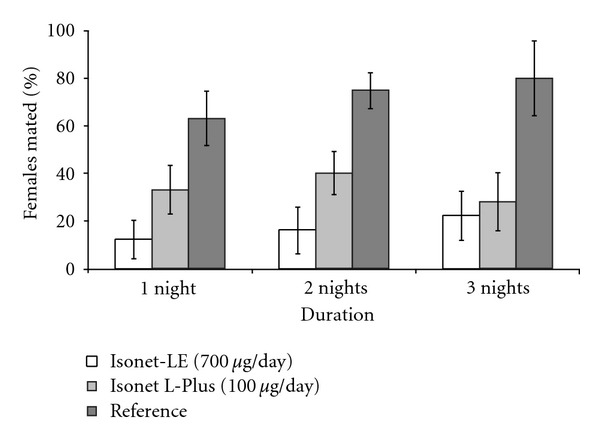
Effect of the duration of insect exposure on the percentage of *E. ambiguella* females mated in field cages (mesh size = 1500 *μ*m, containing no pheromone dispenser). The notation *μ*g/day represents the approximate daily emission rate of pheromone dispensers for *E. ambiguella*; data are untransformed; bars = 1 SE.

92% of *E. ambiguella *females (*N* = 225) exposed were recovered. The three-way ANOVA showed that the date of exposure (*F*_4;  32_ = 10.56, *P* < 0.001) and the pest control scheme (F_2; 32_ = 44.45, *P* < 0.001) had a significant effect on the mating status of females, whereas the duration of exposure (*F*_2;  32_ = 0.38, *P* = 0.684) and the interaction of the control scheme and the duration of exposure did not affect mating (*F*_4;  32_ = 0.82, *P* = 0.522, Figure 3). The proportion of mated females was significantly higher in the reference vineyard than in the two vineyards equipped with pheromone dispensers. Moreover, the more heavily charged Isonet-LE dispensers disrupted mating significantly better than the lower charged Isonet L-Plus dispensers. Overall, one night of insect exposure in our cages appeared to be sufficient to evaluate the effectiveness of pheromone dispensers.

### 3.5. Refinement of the Number of Insects Exposed

**Figure 4:**
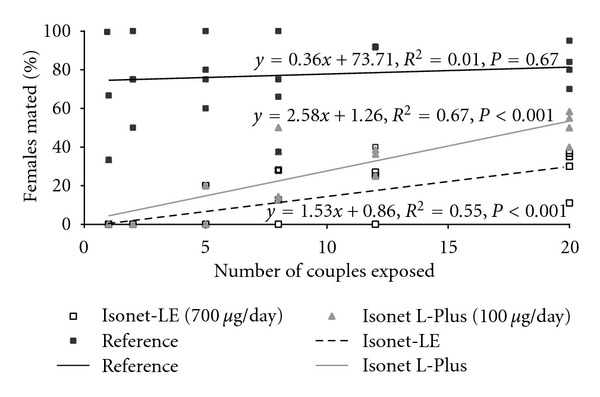
Effect of the number of couples exposed on the percentage of *E. ambiguella* females mated in field cages (mesh size = 1500 *μ*m, containing no pheromone dispenser). The notation *μ*g/day represents the approximate daily emission rate of pheromone dispensers for *E. ambiguella*; data from the three insect control schemes were fitted with linear regression models.

97% of all females of *E. ambiguella* (*N* = 576) exposed were recovered and dissected. The two-way ANCOVA revealed that the date of exposure tended to affect mating and that the number of exposed couples, the pest control scheme, and the interaction of the latter two significantly affected the mating status of females (Table 1). The significant interaction between the number of exposed couples and pest control schemes indicates that the slopes differed significantly among the three pest control schemes (Figure 4). The significant interaction also implies that the two main effects have to be interpreted with caution. Nonetheless, it can be said that the proportion of females mated increased with the number of exposed couples and that significantly fewer females were mated in the two vineyards equipped with pheromone dispensers than in the reference vineyard, in particular at low insect densities. At high insect densities, higher charged Isonet-LE dispensers seemed to disrupt mating better than lower charged Isonet L-Plus dispensers. Overall, the exposure of an intermediate density of eight couples seems to be a good compromise between the ability to identify significant differences between control schemes and an extensive increase in labour.

## 4. Discussion

Our study shows that small insect field cages could constitute a valuable asset for a preliminary evaluation of pheromone dispensers under standardised semifield conditions. The proportion of mated females in cages was significantly reduced in plots equipped with pheromone dispensers compared to untreated sites. Thus, the exposure of a few insect couples for a short period of time can already provide useful insights into the potential efficacy of different mating disruption schemes. Considering the development process outlined here, it should be possible to adapt our cages to other pest systems. Below we first discuss the stepwise development of these insect field cages and then highlight implications of our work for the biotechnological industry.

### 4.1. Development and Refinement of Field Cages

As a first step, we constructed a prototype of a field cage. We then tested if grape moths mate within these cages and if these cages could be suited to measure the effect of sex pheromone dispensing on pest insect mating success. The dissection of females revealed that insects mate without difficulty within the field cages. The cages also allowed to measure significant differences in the mating success of females exposed to sex pheromones compared to controls. This finding was achieved even though the insect density within cages was extremely high and random encounters could be frequent—conditions that are generally assumed to be unfavourable for mating disruption [31]. Nonetheless, our observation is in line with that of others [24, 25] and confirms that mating disruption schemes may reduce insect mating even at high pest densities where random encounters are frequent. A big advantage of our small prototype (0.04 m^3^) compared to the more commonly used larger field cages, for example, 8 m^3^ [15], is that they interfere little with cultural practices such as the passage of tractors and the maintenance of the vine. Secondly, the effectiveness of mating disrupting can be assessed by examination of a female's reproductive organs. This examination provides the advantage of a direct measurement of the impact of pheromone dispensing on mating and not just an indirect effect on mate finding by males as measured by exposing pheromone-baited traps within larger field cages. 

In a second step, we withdrew pheromone dispensers from the inside of the field cage to test if the method would also be suited to measure the effect of prevailing sex pheromones in the vineyard. Unfortunately, mating was less disrupted, indicating that the technique of using a cage without a dispenser placed inside may have limitations. Additional analyses showed that the tissue of 800 *μ*m mesh covering the prototype reduced air flow into cages by almost 90%, whereas a mosquito net of 1500 *μ*m mesh size had 75% air permeability [19]. The tissue was therefore replaced by the more air permeable mosquito net. Thereafter, the proportion of females mated was significantly lower within cages surrounded by pheromone dispensers compared to females similarly exposed in the reference vineyard. Thus, the refined cages with the more permeable mosquito net appeared suitable for measuring the effect of the prevailing sex pheromone regime on mating disruption.

Finally, we refined the duration of insect exposure and the number of insects exposed. Our trials revealed that mating did not significantly increase with the length of exposure of insects within the cages. The exposure of insects of the right age for one night is sufficient to evaluate the effectiveness of different mating disruption schemes in grape moths. However, the proportion of females mated increased with the number of exposed couples. Whereas high densities (>12 couples/cage) demand a considerably greater amount of work in order to rear, expose, and dissect insects, differences between the two pheromone treatments used here were unverifiable at low moth densities (<5 couples/cage). This is in accordance with Vick et al. [32] and Palaniswamy et al. [24] who also observed only small differences between mating disruption schemes at low pest densities. The exposure of about eight couples in the field cages seems therefore to be optimal. This corresponds to a grape moth density of 840′000 couples per hectare (B. Bloesch, personal communication), a pest pressure that is extremely high and that has rarely been observed in commercial vineyards. To conclude, the exposure of eight couples within our field cages during a warm and rainless night allows to compare different pheromone mating disruption schemes targeting grape moths under standardised semifield conditions.

### 4.2. Implications for the Biotechnological Industry

Small insect field cages offer a fast, simple, cheap, and reliable method for a preliminary assessment of pheromone-based control methods. Compared to classical field trials, pest density can be controlled and the minimal size of trial plots can be reduced. Several temporal repetitions can be accomplished over a relatively short period of time, facilitating statistically sound analyses. Furthermore, the effectiveness of newly developed pheromone dispensers can be readily compared to conventional dispensers as well as to untreated plots. However, it should be noted that males cannot follow false plumes within small cages. As a consequence, small insect field cages only allow to evaluate and compare the noncompetitive mechanisms of mating disruption. Thus, findings obtained under these standardised semifield conditions cannot replace the final evaluation of mating disruption schemes in commercial fields, but they may nonetheless help to predict the value of setting up more expensive large-scale field trials.

Following the stepwise development process outlined above, it should be possible to build field cages specifically adapted to different pest systems. Cages are relatively cheap to construct, and the critical step in their development is the determination of cage size and the optimal number of insects exposed. The mesh size of the tissue covering the cage should be chosen as large as possible to assure a maximal air exchange between the cage and the environment, and the duration of insect exposure should be kept not longer than required for a satisfactory degree of mating in the untreated control.

## 5. Conclusions

Small insect field cages provide new perspectives for the assessment of pheromone dispensing on mating and may be of value in applied as well as fundamental research. For example, with these cages, the effectiveness of different formulation types (e.g., sprayable microcapsules, hand-applied dispensers, aerosol puffers) and of new pheromone blends (e.g., plant volatiles) might possibly be evaluated and compared. Secondly, our cages could also be used to test for the minimal density of dispensers required per unit area or the optimal number of point sources in the crop (Kehrli unpublished data). And, thirdly, small field cages may help to investigate the underlying mechanisms of mating disruption in short-distant flight [13, 14, 33]. Thus, small field cages with insect couples offer a simple and fast method for evaluating mating disruption under standardised semifield conditions, and they may therefore be a welcome asset to the biotechnological industry developing this environmental friendly pest control technique.

## Figures and Tables

**Table 1:** ANCOVA table on the effect of date of exposure, number of exposed couples, and mating disruption control scheme on the mating status of *E. ambiguella* females.

Source of variance	df	Sum of Sq	*F* value	*P*
Date of exposure	3	0.20	2.26	0.090
Number of exposed couples (=NEC)	1	0.87	29.37	<0.001
Pest control scheme (=PCS)	2	4.20	70.19	<0.001
NEC*PCS	2	0.31	5.17	0.008
Error	63	1.88		
